# Circulating MicroRNAs Are Not Eliminated by Hemodialysis

**DOI:** 10.1371/journal.pone.0038269

**Published:** 2012-06-08

**Authors:** Filippo Martino, Johan Lorenzen, Julius Schmidt, Mascha Schmidt, Michael Broll, Yvonne Görzig, Jan T. Kielstein, Thomas Thum

**Affiliations:** 1 Institute for Molecular and Translational Treatment Strategies (IMTTS), Hannover Medical School, Hannover, Germany; 2 Department of Medicine, Division of Nephrology and Hypertension, Hannover Medical School, Hannover, Germany; 3 Centre of Clinical and Basic Research, IRCCS San Raffaele, Rome, Italy; University of Sao Paulo Medical School, Brazil

## Abstract

**Background:**

Circulating microRNAs are stably detectable in serum/plasma and other body fluids. In patients with acute kidney injury on dialysis therapy changes of miRNA patterns had been detected. It remains unclear if and how the dialysis procedure itself affects circulating microRNA level.

**Methods:**

We quantified miR-21 and miR-210 by quantitative RT-PCR in plasma of patients with acute kidney injury requiring dialysis and measured pre- and post-dialyser miRNA levels as well as their amount in the collected spent dialysate. Single treatments using the following filters were studied: F60 S (1.3 m^2^, Molecular Weight Cut Off (MWCO): 30 kDa, n = 8), AV 1000 S (1.8 m^2^, MWCO: 30 kDa, n = 6) and EMiC 2 (1.8 m^2^, MWCO: 40 kDa, n = 6).

**Results:**

Circulating levels of miR-21 or -210 do not differ between pre- and post-dialyzer blood samples independently of the used filter surface and pore size: miR-21: F60S: p = 0.35, AV 1000 S p = 1.0, EMiC2 p = 1.0; miR-210: F60S: p = 0.91, AV 1000 S p = 0.09, EMiC2 p = 0.31. Correspondingly, only traces of both miRNAs could be found in the collected spent dialysate and ultrafiltrate.

**Conclusions:**

In patients with acute kidney injury circulating microRNAs are not removed by dialysis. As only traces of miR-21 and -210 are detected in dialysate and ultrafiltrate, microRNAs in the circulation are likely to be transported by larger structures such as proteins and/or microvesicles. As miRNAs are not affected by dialysis they might be more robust biomarkers of acute kidney injury.

## Introduction

MicroRNAs (miRNAs) are a class of endogenous small noncoding RNAs. The single-stranded molecules have a length of 19–23 nt [1]. Gain and loss of function studies revealed that miRNAs play a critical role in the regulation of basically all biological cell functions such as proliferation, differentiation and apoptosis [2]–[8]. MiRNAs are also involved in pathologic pathways of many disease models [6], [9]. Recent studies discovered that miRNAs are detectable in extracellular human body fluids such as blood or urine in a rather stable form [10]–[12].

A potential reason that circulating miRNAs are not degraded by RNAses, is that they are partly included in microvesicles, such as exosomes or bound to protein complexes such as argonaute protein 2 (Ago 2) [13]. Especially in the intensive care unit there is a wide range of filters used for hemodialysis of patients with acute kidney injury. Dialysis membranes do usually not allow passage of larger molecules (>30–40 kDa). However, the dialysis procedure itself might influence the amount of circulating miRNAs. In the present study we therefore analyzed the effect of hemodialysis on circulating miRNA levels in blood and collected spent dialysate in order to investigate, whether the procedure removes miRNAs from circulation. Dialysis membranes of varying pore sizes (degree of molecular weight cut off) were compared.

## Materials and Methods

### Patients and Dialysis unit

**Figure 1:**
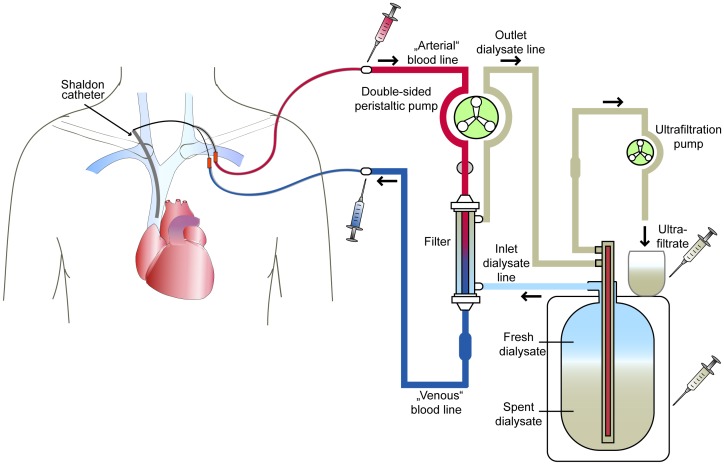
Structure and function of the Genius 75/90 Dialysis System and sample collection. Patient’s blood runs countercurrent to dialysate and enters the dialysis circuit from a Shaldon catheter. The “arterial”, i.e. predialyser blood line (red) passes the filter and blood flows back to the patient via the “venous”, i.e. post-dialyser blood line (blue). Fresh dialysate is prepared individually for every patient’s conditions and stored in a 75 or 90 liter tank. A tube leads the fresh dialysate (light-blue) to the dialysis filter. Exchange of substances and filtration is mediated by an osmotic and pressure gradient and is supported by counter-flow principle. Spent dialysate (grey) flows through the outlet dialysate line back into the tank. Phase formation prevents the mixture of fresh and spent dialysate. An ultrafiltration pump transports the excess filtrate and maintains the pressure gradient of the system. Samples are taken at the marked locations.

The study protocol was approved by the Hannover Medical School Ethics Committee and conducted in accordance with the German Federal Guidelines and the Declaration of Helsinki. Part of this represents a secondary analysis of samples collected for a study published elsewere [14]. Fourteen patients with acute kidney injury (AKI) treated by slow extended daily dialysis (SLEDD) were included (see table 1), using the GENIUS™ dialysis system (Fresenius Medical Care, Bad Homburg, Germany). Its technical details are described elsewhere [15]. In brief, sterile bicarbonate dialysate is filled into a 75- or 90-L tank and is then circulated in a closed loop circuit. During dialysis, fresh dialysate is taken from the top of the tank, whereas the spent dialysate flows back to the bottom. Eight patients of the collective were treated only with the regularly used filter system (polysulfone high-flux dialyzer (F60S), 1.3 m^2^ effective surface area, inner lumen 200 µm, wall thickness 40 µm, Fresenius Medical Care, Molecular weight cut off (MWCO): 30 kDa). Two of them received a second dialysis session at a later point in time which let us classify these results as an independent event. For further validation we recruited six more patients that received a dialysis session with two other different filter systems. The EmiC 2 (1.8 m^2^ effective surface area, inner lumen 220 µm, wall thickness 35 µm, polysulfone, Fresenius Medical Care) has an enhanced middle molecule clearance and therefore among other things a higher MWCO of 40 kDa. We tested whether a larger pore size might result in depletion of circulating miRNAs. The AV 1000 S dialysis filter system (Fresenius Medical Care) effective surface area 1.8 m^2^, inner lumen 220 µm, wall thickness 35 µm; allowing elimination of substances with a molecular weight of up to 30 kDa. The dialysate and countercurrent blood flow ranged between 150 to 165 mL/min in all dialysis sessions. Samples from pre-dialyser and post-dialyser blood line directly before and after the dialysis filter were collected 30 minutes after starting the dialysis session. Spent dialysate and ultrafiltrate samples were collected after the end of treatment (Duration: 490 min (480 min to 630 min)). The system, dialyser and sampling ports are described in figure 1.

**Table 1:** Demographic and clinical patient characteristics.

	Total	F60S	EMiC 2/Ultraflux AV 1000 S	p-value
**Number of Patients:** **Male (n; %)** **Female (n; %)**	148; 57.1%6; 42.9%	84; 50.0%4; 50.0%	64; 66.7%2; 33.3%	0.8
**Age (years)**	49 (36 to 59)	53 (41 to 59)	40 (32 to 62)	0.7
**BMI**	25.7 (20.8 to 29.2)	24.6 (20.8 to 25.7)	31.5 (23.0 to 30.9)	0.4
**Residual renal function (ml/d)**	0 (0 to 0)	0 (0 to 0)	28 (22 to 33)	0.8
**Urea (mmol/l)**	15.4 (8.5 to 23.7)	13.4 (7.8 to 29.7)	18.6 (9.9 to 23.9)	0.7
**APACHE II Score**	23 (20 to 28)	22 (21 to 26)	27 (16 to 29)	0.9

BMI, Body mass index; APACHE II, Acute Physiology and Chronic Health Evaluation.

### RNA Isolation

RNA isolation was performed with plasma, ultrafiltrate and dialysate samples by using the MasterPure RNA Purification kit (Epicenter Technologies) according to the instructions of the manufacturer. In addition, we supplemented the samples with 1 µL of 3 fmol/µL *Caenorhabditis elegans* miR-39 (cel_miR-39) to normalize results.

### Detection and Quantification of miRNAs

Isolated RNA was reverse transcribed with hsa_mir-21, hsa_miR-210 and cel_miR-39 primers using the TaqMan microRNA Reverse Transcription kit (Applied Biosystems, Foster City, CA) according to the manufactureŕs advices. Then both miRNAs were detected and validated by quantitative real-time PCR (qRT-PCR) via TaqMan MicroRNA Assays (Applied Biosystems). Levels of circulating miRNA were normalized to cel_miR-39 which was spiked in as external control.

### Statistical Analysis

All statistics were performed with Graph Pad Prism 4. A nonparametric two-tailed Mann-Whitney test was used for statistical analysis of two groups. Data are represented as median and lower and upper quartile, if not described differently.

## Results

**Figure 2:**
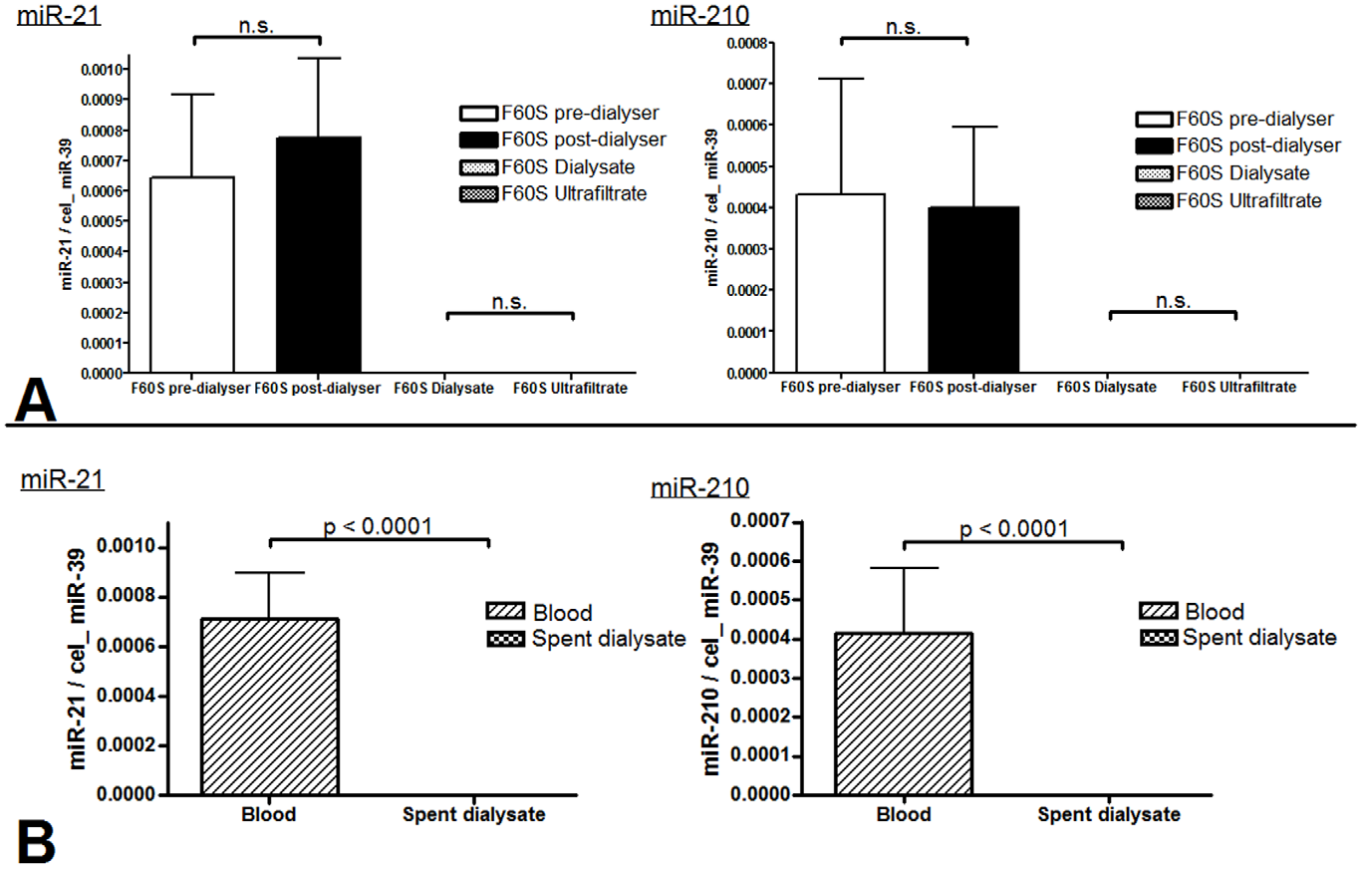
Levels of circulating miR-21 and -210 in different sample types with the F60S dialysate filter. Ten independent dialysis sessions in eight different patients were analyzed for the F60S filter system. MiR-21 and miR-210 levels are not significantly different between pre-dialyser and corresponding post-dialyser blood lines (miR-21: p = 0.35; miR-210: p = 0.91). Also the difference between spent dialysate and ultrafiltrate is not of statistical significance (miR-21: p = 0.80; miR-210: p = 0.74). However, averaged values of blood side versus spent dialysate reached high statistical significance for both microRNAs with p-values of <0.0001. Data are represented as mean and standard error of the mean.

Patients with acute kidney injury undergoing SLEDD using a F60S filter, we did not identify any effect of the dialysis procedure on circulating miRNA-21 and -210 levels comparing pre-dialyser and post-dialyser blood samples (miR-21: p = 0.35; miR-210: p = 0.91 (Figure 2A)). In line with this only traces of miR-21 and miRNA-210 could be found in both, dialysate and ultrafiltrate (miR-21: Dialysate: 1.6×10^−6^ (3.0×10^−7^ to 2.8×10^−6^), ultrafiltrate: 1.3×10^−6^ (3.4×10^−7^ to 2.4×10^−6^); miR-210: dialysate: 1.0×10^−7^ (2.8×10^−8^ to 4.5×10^−7^), ultrafiltrate: 8.3×10^−8^ (3.0×10^−8^ to 2.1×10^−7^)). Also there was no difference between dialysate and ultrafiltrate (miR-21: p = 0.80; miR-210: p = 0.74 (Figure 2A)). As shown in Figure 2B miRNA values in blood within the F60S system for miR-21 and miR-210 were significantly higher than in dialysate/ultrafiltrate (p<0.0001) suggesting only minimal passage of miRNAs throughout the dialyser membrane.

**Figure 3:**
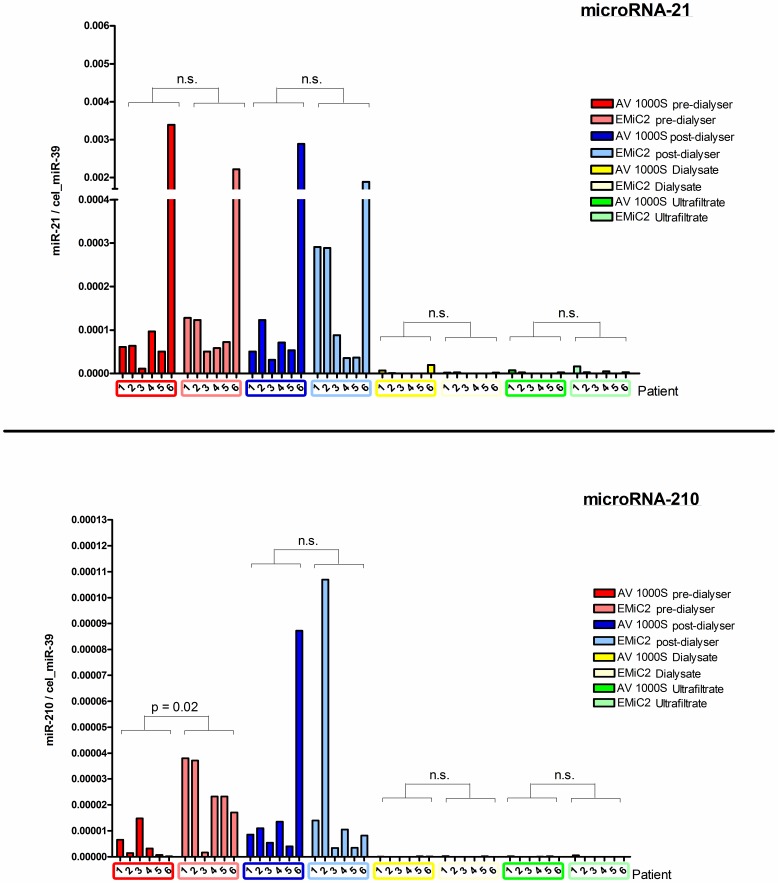
Comparison of influence of two different filter properties on circulating microRNA levels. Six patients received at least one dialysis session with each of the two filter systems AV 1000 S and EMiC 2. Circulating levels of miR-21 and -210 were not differently altered by the two different dialyser properties. (miR-21: AV1000S pre-dialyser vs. EMiC 2 predialyser p = 0.59, post-dialyser p = 0.70, dialysate p = 0.94, ultrafiltrate p = 0.39; miR-210: pre-dialyser p = 0.02, post-dialyser p = 0.82, dialysate p = 0.59, ultrafiltrate p = 0.31) Only pre-dialyser line showed a significant alteration, which is not of relevance for our main conclusion. Data are represented as mean and standard error of the mean.

Also dialysers with a larger surface area and pore size (Fresenius AV 1000 S 1.8 m^2^ (MWCO: 30 kDa), EMiC 2 1.8 m^2^ (MWCO: 40 kDa)) had no significant effect on circulating miRNA levels (miR-21: AV1000S pre-dialyser vs. EMiC 2 pre-dialyser p = 0.59, post-dialyser p = 0.70, dialysate p = 0.94, ultrafiltrate p = 0.39; miR-210: pre-dialyser p = 0.02, post-dialyser p = 0.82, dialysate p = 0.59, ultrafiltrate p = 0.31) (figure 3). The significant difference between the pre-dialyser line of AV 1000 S versus EMiC2 in miRNA-210 levels cannot be caused by different filter properties and is therefore not relevant for our main conclusion.

Also, the difference of miRNA-21 and -210 levels in blood versus spent dialysate levels using the EmiC 2 or AV 1000 S filter showed similar results for both miRNAs as previously shown for the F60S dialyser (p<0.0001; data not shown). Difference in miR-21 and -210 depletion ratios between the predialyser blood line and spent dialysate for AV 1000 S (miR-21: 2.2×10^−3^ (3.6×10^−4^ to 7.9×10^−2^); miR-210: 7.3×10^−3^ (3.0×10^−3^ to 3.3×10^−1^)) and EMiC 2 (miR-21: 1.2×10^−2^ (5.9×10^−4^ to 1.1×10^−1^); miR-210: 4.2×10^−3^ (8.3×10^−4^ to 1.1×10^−2^)) were not different (miR-21: p = 0.70; miR-210: p = 0.31 (data not shown)). This implicates that the different filter properties does not significantly alter circulating levels of at least miRNA-21 and -210.

Anticoagulation with heparin had no effect on the detectable miRNA levels in this study. Neither miR-21 (non-heparin treated pre-dialyser vs. heparin treated pre-dialyser p = 0.49, post-dialyser p = 1.00) nor miR-210 (non-heparin treated pre-dialyser vs. heparin treated pre-dialyser p = 0.19, post-dialyser p = 0.98) showed a significant difference in the entire patient collective between heparin treated and non heparin treated group.

## Discussion

This is the first study, which describes the influence of hemodialysis therapy on circulating miRNA levels in patients with AKI. The salient finding is that miRNA, although only small in size, are not removed by various dialyser membranes, even those designed to remove middle-sized molecules.

MiRNAs interfere with a variety of pathologic pathways [6], [16], [17]. MiRNA-21 is a crucial player in various disease models such as cancer, heart failure or fibrosis in heart, lung or kidney [8], [18]–[23]. Furthermore published evidence implicates that miR-21 is important in immune cell development and function [24], [25]. Patient number 6 showed relatively high values of circulating miRNAs. Interestingly, this patient was involved in complex disease processes such as hepatitis B and C co-infection which led to liver transplantation. It is possible that these circumstances induced high levels of circulating miR-21. In fact there is a growing body of evidence that this miRNA is elevated in the circulation of patients with hepatitis C and therefore might be a potential biomarker [26]. MiR-210 has beenshown to be involved in kidney disease such as T-cell mediated rejection or clear cell carcinoma [16], [27]. Furthermore it is a strong predictor of survival in critically ill patients with acute kidney injury in plasma [9], [16].

Recent studies suggest that circulating miRNAs in general might have biosignaling functions and are transported by microvesicles and/or proteins [12], [28], [29]. Therefore we hypothesized that a dialysis procedure could remove miRNAs at least partially from blood, which lead to altered circulating levels of these small ribonucleotides of the circulation. This might have biological consequences. Surprisingly, we could not find a significant alteration of miRNAs in plasma after passing the dialysis filters. This leads to the conclusion that dialysis therapy does not deplete patient’s blood of possibly biologically active miRNAs in the circulation. However, small amounts of miRNAs were detectable in the ultrafiltrate as well as in spent dialysate suggesting that a small portion of miRNAs in the blood is transported by very small structures or as a free unbound form.

There are several possibilities of stabilizing circulating miRNAs in the blood; two main transporter systems have been identified. MiRNAs are transported in microvesicles and/or exosomes, apoptotic bodies and other microparticles [30]. In addition miRNAs are transported by non-vesicle associated protein and lipoprotein complexes [13]. Turchinovich et al. showed that the majority of circulating miRNAs are bound to a molecular family called argonaute proteins [31]. Especially Ago 2, a 97 kDa member of the RNA-induced silencing complex (RISC), is the most frequent miRNA carrier [32]. Furthermore, this group suggests that other transport forms such as exosomes may play only a minor role and more than 97% of the miRNAs are not exosome-associated [31]. However our study only permits the conclusion that miRNAs must be mainly transported by RNA-binding proteins (>30–40 kDa), microvesicles and/or other structures larger than 30–40 kDa.

**Figure 4:**
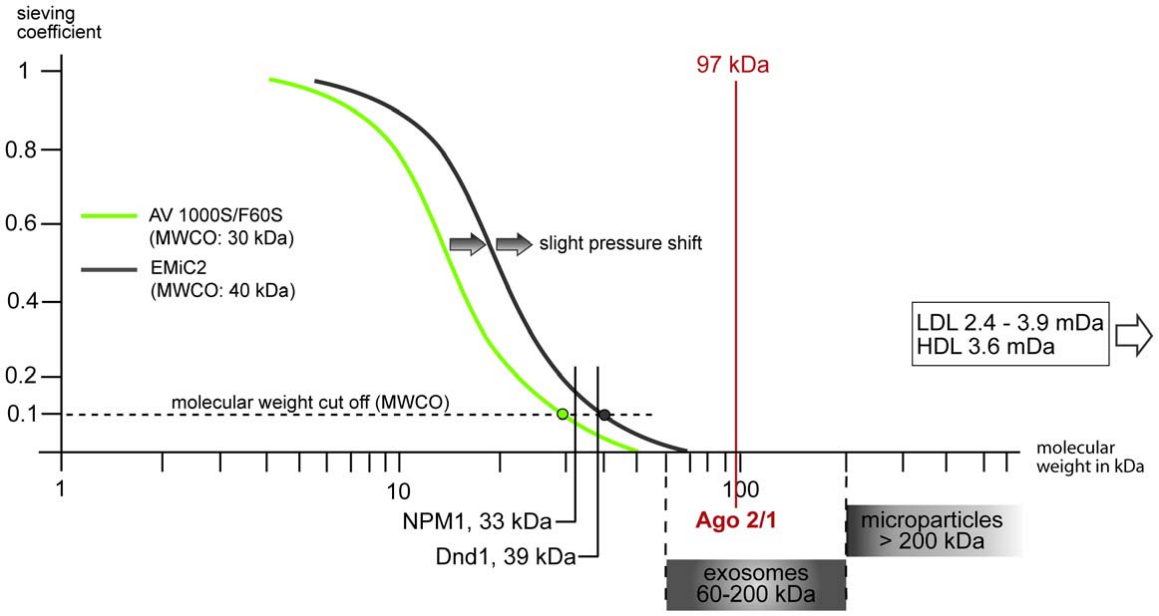
Filter-characteristics and potential microRNA carriers. The figure shows the filter properties characterized by their sieving coefficients depending on the molecular weight of the possibly passing molecules. Sieving coefficient is the ratio between passing and retained mass of molecules of a defined molecular weight. Molecular weight cut off (MWCO) is a special indicator to describe filter-characteristic. It characterizes the weight of molecules which are ∼ 90% retained and therefore negligibly passing the filter. A slight pressure dependent shift towards higher MWCOs is possible. Furthermore there are marked various known microRNA carriers with their molecular weight. NPM1, Nucleophosmin 1; Dnd1, Dead end 1; Ago2/1, Argonaute protein 2/1; LDL, Low density protein; HDL, High density protein.

Previous studies demonstrated unbound/naked miRNAs not to be protected against the endogenous RNAses in the extracellular fluids and may therefore be very unstable [13]. That makes it implausible that the detectable traces of miR-21 and -210 in ultrafiltrate and dialysate are not accompanied by stabilizing factors. Recent investigations describe two RNA-binding proteins that possibly could be candidates for this passage, namely nucleophosmin 1 (NPM 1) and dead end 1 (Dnd 1) [33]. As shown in figure 4 NPM 1 and Dnd 1 are the only known miRNA-carriers which are small enough to permeate at least the EMiC2 filter. Here it should be noted that filter-characteristics depend on pressure differences between the blood and effluent circuit. Because of this slight pressure dependent shift of the sieving coefficient curves towards a little higher molecular weight cut offs, it could be possible that very small exosomes might pass the dialyser as well. All other known transport forms such as Low and High density protein (LDL/HDL), microvesicles or even Ago 2 are too large to penetrate the membrane effectively. Thus, these structures are likely the major transporters of miRNAs. A recent study in chronic hemodialysis patients suggested that levels of miR-499 are in fact eliminated by hemodialysis [34]. This finding thus runs contradictory to the results presented here. In order for miRNAs to be eliminated by hemodialysis they have to be either bound to very small molecules or circulate unbound in blood. The study by Mitchell and coworkers suggests that “naked” (i.e. unbound) miRNAs are degraded within less than 2 minutes after addition to human plasma [11]. In addition, as figure 4 suggests only Dnd1 or NPM1 are able to pass the EMIC2 filter system. Based on this we do not believe that circulating miRNAs can be altered by the hemodialysis procedure. Thus, an alteration of miR-499 levels in chronic hemodialyis patients might rather be due to other factors (i.e. degradation). However, different miRNAs have been shown to be transported by varying means [35]. The mirRNA let-7a was shown to be mainly detected in microvesicles, while others were associated with Ago2 (e.g. miR-16 and miR-92a) [35]. Thus, miRNA packaging into microvesicles or RNA binding proteins likely follows a cell type-specific expression and/or release pattern. It is therefore conceivable that miR-499 is subjected to different kinetics. In pre-dialysis patients with chronic kidney disease the levels of circulating miRNAs have been shown to be reduced suggesting altered miRNAs kinetics depending on renal function [36]. However, our results suggest that the hemodialysis technique as such does not influence levels of circulating miRNAs.

Based on our results we concluded, that: (I) miRNAs are likely to be carried and stabilized by larger structures (e.g. RNA-binding proteins and or microvesicles) (II) These structures must have a molecular weight not less than 30 respectively 40 kDa and (III) a small but detectable amount of miRNAs is carried in smaller structures with a kDa of less than 30 or is freely available in the blood.
